# Magnetic field-guided enhancement of nanoparticle uptake and hyperthermia efficacy in 3D tumour models

**DOI:** 10.1039/d6na00172f

**Published:** 2026-08-20

**Authors:** Lilianne Beola, Lucía Gutiérrez, Raluca M. Fratila, Jesús M. de la Fuente, Laura Asín, Valeria Grazú

**Affiliations:** a Instituto de Nanociencia y Materiales de Aragón (INMA), CSIC-Universidad de Zaragoza C/Pedro Cerbuna 12, 50009 Zaragoza 50018 Spain lbeol@unizar.es valeria.grazu@csic.es; b Centro de Investigación Biomédica en Red de Bioingeniería, Biomateriales y Nanomedicina (CIBER-BBN) 50018 Zaragoza Spain

## Abstract

Magnetic hyperthermia (MH) using iron oxide nanoparticles (MNPs) is a promising cancer treatment strategy, yet its efficacy strongly depends on efficient intracellular nanoparticle delivery. Here, we compared two strategies to enhance MNP uptake by tumour cells: (i) increasing the administered MNP dose and (ii) applying a static magnetic field (MF) gradient to promote nanoparticle-cell interactions and increase availability at the cell surface. Human tumour cell lines, representing distinct challenges for nanoparticle internalisation due to differences in growth behaviour and cellular organisation, were first incubated with glucose-functionalised 11 nm MNPs under two-dimensional (2D) culture conditions to control and quantify intracellular uptake under each strategy. Cells with pre-internalised MNPs were then embedded in collagen-based three-dimensional (3D) tumour models, where MH was applied and therapeutic efficacy was evaluated by confocal microscopy and flow cytometry. While both strategies increased intracellular MNP levels, only MF-assisted internalisation resulted in effective MH-induced cytotoxicity under mild conditions across all tested cell lines. These findings indicate that antitumour MH efficacy is not solely determined by nanoparticle uptake, but also by the strategy used to enhance internalisation, revealing an additional layer of complexity for designing efficient MH protocols.

## Abstract 2

Magnetic hyperthermia (MH) using iron oxide nanoparticles (MNPs) is a promising cancer treatment strategy, yet its efficacy strongly depends on efficient intracellular nanoparticle delivery.

## Graphical abstract

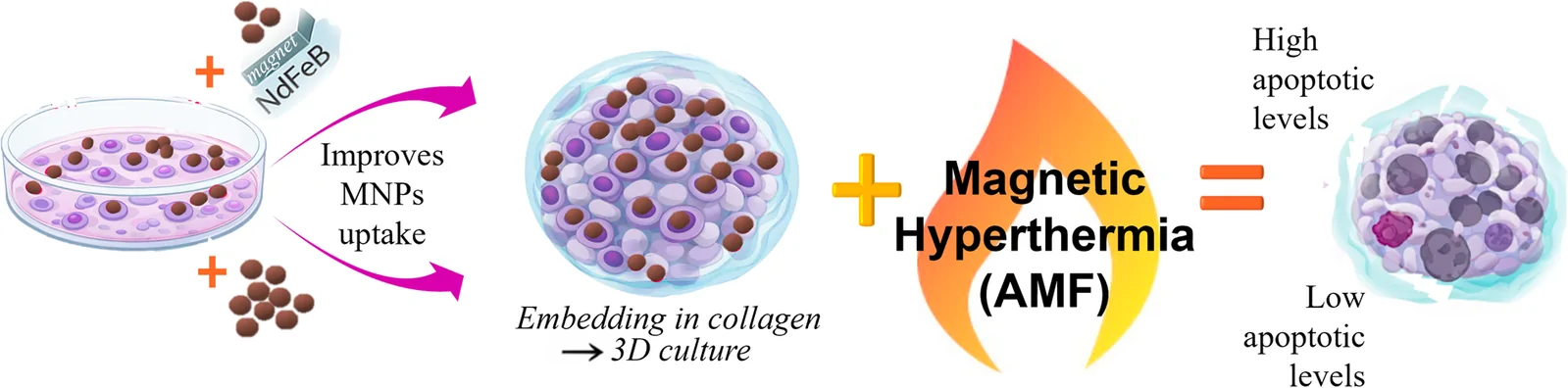

## 1 Introduction

Magnetic hyperthermia (MH) is a developing therapy for cancer^1^ that involves exposing magnetic nanoparticles (MNPs), previously accumulated within the tumour, to an external alternating magnetic field (AMF). Under AMF stimulation, the magnetic moments of the nanoparticles rotate, leading to heat generation through several physical mechanisms.^2–4^ This localized heating interferes with cancer cell proliferation and differentiation,^5–9^ destabilises tumour structure and microenvironment at the tissue level,^11^ and induces molecular changes that trigger tumour cell death.^12–15^ MH has been proposed as a complementary, adjuvant, or concomitant therapy in oncology and is also under investigation for pre- and post-surgical applications.^16–21^ Despite promising preclinical and clinical advances, some limitations remain before MH can be fully established as a standard clinical procedure.^10,18,22^

One of the major challenges in antitumoural MH is achieving sufficient intracellular MNP accumulation to reach a therapeutic response under an AMF, while avoiding disruption of cellular homeostasis.^10,13,23,24^ Insufficient uptake limits heat generation and therapeutic efficacy.^10,25,26^ Conversely, excessive intracellular MNP accumulation can induce lysosomal dysfunction, oxidative stress, and iron imbalance, even in the absence of an AMF.^13,23,24^ These effects compromise remote spatiotemporal control of the treatment.

To address this, several strategies have been explored to regulate MNP internalisation. More complex approaches include membrane permeabilization^27^ and the incorporation of MNPs into delivery systems such as liposomal,^28^ polymeric,^29^ or extracellular vesicle-based carriers^30^ to enhance cellular uptake. While these methods can improve internalisation, their translational potential is often limited by cytotoxicity, modest efficiency, scalability challenges, and procedural complexity.^31–33^

In contrast, more straightforward strategies rely on modulating the local nanoparticle concentration to achieve intracellular levels sufficient for therapeutic heat generation. In this way, MNP internalisation can be controlled either by increasing the total administered dose,^10,13,34^ or by using static magnetic field (MF) gradients^35–39^ to promote nanoparticle accumulation at the cell surface, thereby enhancing the effective dose close to the cell membrane. While escalating the global dose is a common approach,^10,13,34^ physical magnetic assistance offers a means to drive uptake even at low concentrations. This principle is already successfully exploited in magnetofection, where MF gradients, typically generated using Nd–Fe–B permanent magnets,^36^ enhance cellular internalisation *via* clathrin- and caveolin-mediated endocytic pathways.^40–42^ External magnetic field-based targeting has also been applied in magnetic drug targeting frameworks, where spatially controlled magnetic gradients have been used to investigate enhanced local accumulation and retention of magnetic carriers in target tissues.^43–48^ Despite this demonstrated ability to enhance cellular uptake, utilizing static MF gradients to reach hyperthermia-relevant intracellular loads while maintaining low global MNP doses remains relatively underexplored. Furthermore, nanoparticle internalisation is strongly influenced by tumour type and cellular phenotype; consequently, relying on a single physical or biochemical mechanism often falls short when confronting the heterogeneous nature of different tumour models.

To address these limitations, a practical alternative lies in combining biochemical targeting with physical magnetic guidance. Building upon our previous findings, where modulation of the administered dose of glucose-functionalised MNPs produced high MH efficiency in the human pancreatic tumour MIA PaCa-2 cell line both *in vitro* and *in vivo*,^10^ here we further explore the effect of coupling this carbohydrate functionalisation with static MF gradients. The glucose coating provides an active metabolic targeting component that exploits the elevated glucose demand characteristic of tumour cells,^49–51^ thereby promoting enhanced nanoparticle-cell interactions and facilitating cellular internalisation.^49,52–54^ The combination of both strategies can synergistically enhance nanoparticle association and promote more consistent intracellular accumulation across diverse cellular phenotypes without requiring excessive global doses.

Accordingly, this study evaluates the impact of global dose escalation *versus* static MF gradient-assisted uptake on MH efficiency across distinct tumour cell lines *in vitro*. To examine how cell line-dependent variations influence hyperthermia outcomes, HCT-116 colorectal cancer and MCF-7 breast adenocarcinoma cells were evaluated alongside MIA PaCa-2. Our previously reported *in vitro* data for MIA PaCa-2,^10^ generated in parallel with the current experiments, serve as a reference framework; their high metabolic activity and robust endocytic capacity establish them as a well-characterised baseline for comparison.^55–57^ In contrast, HCT-116 cells feature tight intercellular junctions and defined structural traits that modulate nanoparticle penetration, whilst also exhibiting chemoresistance and specific apoptosis-related phenotypes relevant for therapeutic evaluation.^58–61^ Conversely, MCF-7 cells exhibit multidrug resistance and unique membrane dynamics that alter internalisation and intracellular trafficking.^62–65^ Their tendency to grow as compact clusters in culture restricts membrane accessibility and limits nanoparticle uptake,^27,65,66^ offering a challenging, complementary context. These variations in morphology, structural organisation, and growth patterns ultimately influence nanoparticle interactions and internalisation behaviour.^67–69^ Incorporating distinct cellular models thus enables a comprehensive assessment of strategy robustness and potential applicability across diverse tumour microenvironments.

The strategies were first evaluated in 2D cultures to optimise nanoparticle preloading conditions and quantify cellular uptake across the selected cell lines. Preloaded cells were then embedded in three-dimensional (3D) collagen-based matrices and exposed to an AMF to assess the therapeutic efficacy of MH (Scheme 1). This 2D-to-3D approach, established in our previous work,^10^ not only enables the evaluation of hyperthermia under more biologically relevant conditions but also ensures a fixed, stable cellular magnetic load throughout treatment.^10,13,70^ This stability is achieved by the removal of non-associated MNPs through extensive washing prior to collagen encapsulation. Consequently, MNPs associated with cells are maintained during AMF exposure, enabling the observed MH effects to be primarily attributed to cell-bound nanoparticles.

**Scheme 1:**
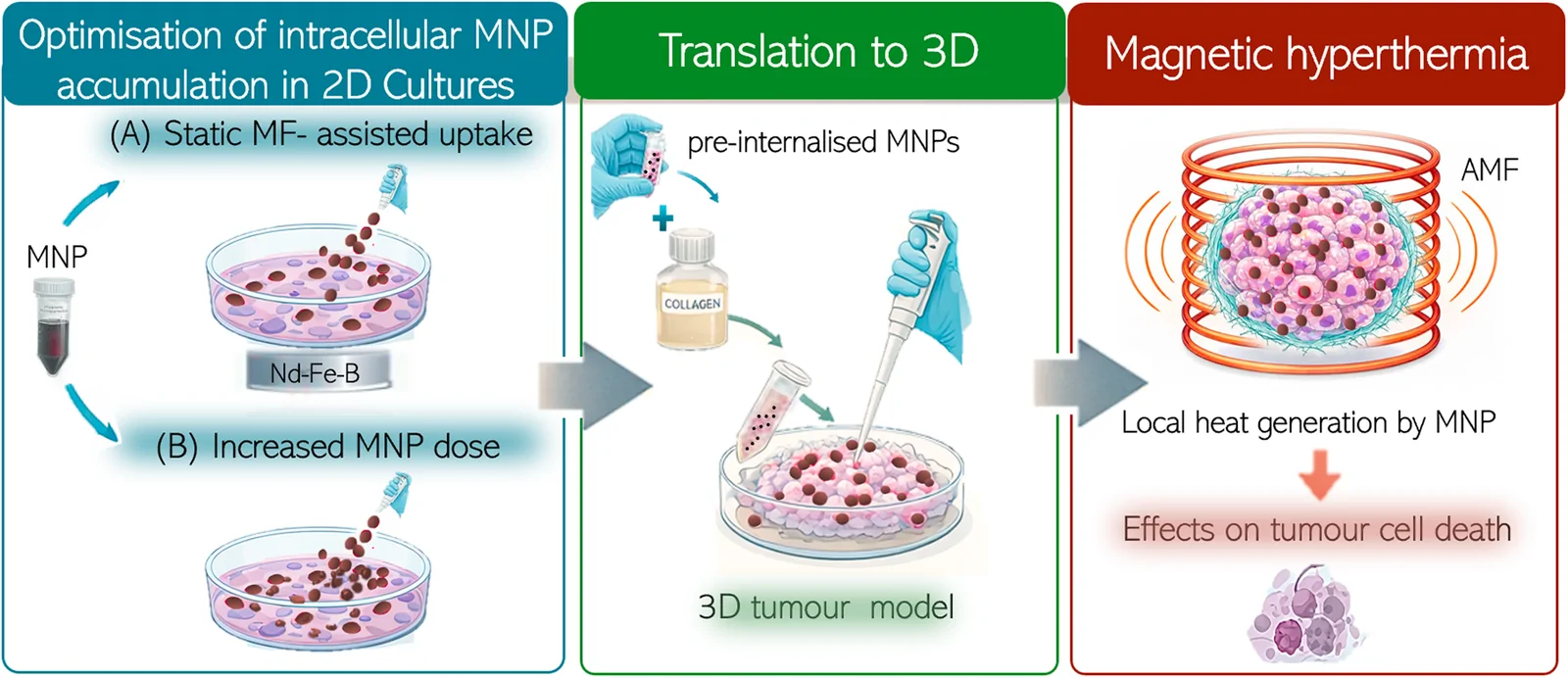
Schematic illustration of the experimental strategy. Intracellular MNP accumulation was optimised in 2D cell monolayer cultures using static magnetic field (MF) gradient-assisted uptake or increased MNP dose, followed by translation to a hydrogel-based 3D tumour model for evaluation of magnetic hyperthermia (MH) efficacy under an alternating magnetic field (AMF).

Our results demonstrate that, although both strategies enhance cellular uptake, magnetic hyperthermia effectiveness is strongly influenced by the method used to enhance nanoparticle internalisation rather than intracellular nanoparticle levels alone. This finding reflects a complex interplay between uptake dynamics and therapeutic outcome. While the specific mechanisms underlying these differences were not investigated in this study, the results highlight the importance of the uptake-enhancing methodology as a key determinant of MH efficacy. These considerations may be relevant when designing and interpreting magnetic hyperthermia experiments across *in vitro* and *in vivo* settings and may inform the selection of uptake-enhancing strategies in future studies.

## 2 Materials and methods

### 2.1 Magnetic nanoparticles: synthesis, functionalization and characterization

Iron oxide nanoparticles (MNPs, 11 nm) were synthesised by thermal decomposition in an organic medium and transferred to water *via* coating with amphiphilic polymer PMAO (poly(maleic anhydride-*alt*-1-octadecene), MW 30 000–50 000 Da) functionalised with the fluorophore TAMRA (tetramethylrhodamine 5(6)-carboxamide cadaverine; Anaspec, Seraing, Belgium; *λ*_ex_ = 543 nm, *λ*_em_ = 570 nm). The MNPs were subsequently functionalised with glucose (4-aminophenyl β-d-glucopyranoside) following reported procedures.^10,13,70^ After 3 h at room temperature, excess glucose was removed by centrifugal washing with phosphate-buffered saline (PBS, pH 7.4). The nanoparticles were resuspended in PBS and sterilised by 0.22 µm filtration (Merck Millipore).

Functionalisation was confirmed by agarose gel electrophoresis (1% agarose, 0.5× TBE (Tris–Borate–EDTA), 90 V, 30 min), imaged using a Bio-Rad Gel Doc EZ system as previously reported.^10,13,70^ Relative mobility (*R*_f_) was calculated as migration distance/gel length and quantified using Gel Analyzer 2010. Iron concentration was determined by a standard colourimetric assay.^71^

Particle size and morphology were analysed by transmission electron microscopy (TEM) using a Tecnai T20 microscope (FEI, USA) operating at 200 kV. Samples were prepared by depositing diluted MNP suspensions onto carbon-coated grids and allowing them to dry prior to imaging. Particle size was determined by manual analysis of at least 200 nanoparticles using Digital Micrograph software. Dynamic light scattering (DLS) and *ζ*-potential were measured in water, PBS and complete Dulbecco's Modified Eagle's Medium GlutaMAX Supplement (cDMEM (Gibco, Thermo Fisher Scientific) 10% foetal bovine serum (FBS; Invitrogen)) using a Malvern Zetasizer Nano-ZS (10 runs, five replicates per measurement, 25 °C, pH 7), confirming functionalisation efficiency and colloidal stability.

Heating capacity was evaluated using a DM100 alternating magnetic field system (Nanoscale Biomagnetics, Spain). A suspension (1 mg_Fe_ mL^−1^) was exposed to *H* = 20 kA m^−1^ at *f* = 829 kHz for 5 min, and temperature was recorded using an integrated optical fibre sensor. The specific loss power (SLP) was calculated from the initial slope of the heating curve according to: SLP = *Cm*^−1^*V*_s_ (Δ*T*/Δ*t*); where *C* is the specific heat capacity of the sample, including both the magnetic material and the dispersion medium, *m* is the mass of magnetic material, *V*_s_ is the sample volume, and Δ*T*/Δ*t* corresponds to the initial slope of the temperature–time curve. Magnetic performance was characterized by field-dependent magnetization (*M*(*H*)) using a Quantum Design MPMS-XL SQUID magnetometer (USA). *M*(*H*) hysteresis loops were recorded at 300 K under a maximum applied field (*H*) of up to 5 T.

### 2.2 Cell lines and cell culture

The human breast cancer cell line MCF7 (ATCC® HTB-22™), the human pancreatic cancer cell line MIA PaCa-2 (ATCC® CRL-1420™), and the human colorectal carcinoma cell line HCT 116-Luc2 (ATCC® CCL-247-LUC2™) were cultured and maintained in cDMEM with GlutaMAX™ Supplement (Gibco®, Thermo Fisher Scientific) or complete McCoy's 5A medium supplemented with 25 mM 4-(2-hydroxyethyl)-1-piperazineethanesulfonic acid (HEPES) and GlutaMAX™ (cMcCoy's 5A; BioWhittaker™, Lonza), as appropriate. Complete media were supplemented with 10% FBS (Invitrogen), 100 U mL^−1^ penicillin G (sodium salt), and 100 µg mL^−1^ streptomycin sulfate (Invitrogen). Cells were maintained at 37 °C in a humidified atmosphere containing 5% CO_2_ in a water-jacketed incubator (NU 4500E; NuAire Inc., MN, USA). Depending on confluency, cultures were passaged every 4–5 days at a 1 : 10 dilution. For cell detachment, cultures were incubated with trypsin–EDTA solution (Sigma-Aldrich) for 3–5 min at 37 °C, and cells were subsequently collected in fresh cDMEM or cMcCoy's 5A medium, respectively.

#### 2.2.1 Generation of three-dimensional cell culture models

To obtain the 3D cell culture, a previously reported protocol was followed.^10,13,70^ Cells from the different lines were incubated under monolayer conditions with magnetic nanoparticles (MNPs; 100 or 250 µg_Fe_) in a final volume of 0.25 mL of complete culture medium at 37 °C in a humidified atmosphere containing 5% CO_2_ for 24 h. During incubation, cells were either exposed or not exposed to a magnetic field gradient generated by a cylindrical neodymium–iron–boron (Nd–Fe–B) disc magnet (6 × 3.6 mm, diameter × height; nickel-plated; ±350 mT; ref. N35D636; Laboutiquedeliman®, Barcelona, Spain) positioned under each well of a 24-well plate. The magnet produced a non-uniform field across the culture, inducing a magnetic force on the MNPs and promoting particle guidance towards the cells.

Following incubation, the medium containing MNPs was removed, and cells were washed and subsequently detached using Trypsin–EDTA solution (Sigma-Aldrich). Cells were then washed twice by centrifugation (161×*g*, 5 min) and embedded in rat tail collagen type I (First Link, UK) to generate 3D cultures. After gelation (∼20 min), 0.5 mL of complete cDMEM or cMcCoy's 5A medium was added to the respective constructs, which were maintained at 37 °C for 2.5 h prior to experiments to allow stabilisation of the cells within the 3D matrix.

### 2.3 MNP toxicity, cellular localisation and uptake assays

#### 2.3.1 MTT assay

Cells (1 × 10^4^ per well) were seeded in 96-well plates in a final volume of 0.25 mL per well for each cell line of interest (MCF7, HCT 116, and MIA PaCa-2). After 24 h, the complete culture medium was gently removed. Different amounts of MNPs (50, 100, 150, and 250 µg_Fe_) were added to the cells in a final volume of 0.25 mL and incubated for 24 h. Three biological replicates were prepared for each concentration. Following incubation, the supernatant was discarded, the cells were washed with PBS, and 3-(4,5-dimethyl-2-thiazolyl)-2,5-diphenyl-2*H*-tetrazolium bromide (MTT) solution (0.25 mg mL^−1^) was added to each well in a final volume of 0.2 mL. Plates were incubated at 37 °C in a humidified atmosphere containing 5% CO_2_ for approximately 2 h until formazan crystal formation was observed, then centrifuged at 2500 rcf for 25 min to sediment the cells. The supernatant was removed, and the formazan crystals were dissolved by adding 0.1 mL of dimethyl sulfoxide (DMSO). Absorbance was measured at 550 nm.

#### 2.3.2 Optical microscopy

Bright-field images of cells incubated with MNPs under different conditions were acquired prior to embedding in collagen-based 3D models. Cells adhered to 24-well culture plates were observed using an Eclipse TE2000 inverted microscope (Nikon Instruments Inc., Melville, NY, USA). Images were acquired using a 10× objective under bright-field illumination. The exposure time was kept constant for all conditions (standard exposure settings of the microscope software) to ensure comparability between samples. Identical acquisition settings were used across all experimental conditions within each experiment.

#### 2.3.3 Confocal laser scanning microscopy and 3D reconstruction

MNP localisation within the 3D cell cultures was visualised using a Zeiss LSM 880 confocal laser scanning microscope equipped with a 63×/1.40 Oil Plan-Apochromat objective. Prior to imaging, collagen gels were fixed for 20 min with 4% paraformaldehyde (PFA, Thermo Scientific Chemicals) at room temperature, followed by nuclear counterstaining with DAPI (4′,6-diamidino-2′-phenylindole dihydrochloride, Thermo Scientific™) and actin cytoskeleton staining with Phalloidin-488 (Invitrogen™). To capture the multi-channel fluorescence signals, the following excitation and detection settings were utilised: DAPI (nuclei) was excited at 405 nm (diode laser) with detection at 410–460 nm; Phalloidin-488 (cytoskeleton) was excited at 488 nm (argon ion laser) with detection at 495–550 nm; and TAMRA-labelled MNPs were excited at 561 nm (DPSS laser) with detection at 570–640 nm. Additionally, MNPs were visualised using confocal reflection mode (direct laser backscattering) alongside fluorescence to confirm their intracellular localisation and structural integrity within the 3D gel matrix. Laser power was systematically kept below 2.0% across all channels to prevent photobleaching. Images were acquired at a scan speed of 6 (pixel dwell time of ≈2.0 µs) with the pinhole set to 1.0 Airy Unit (AU) for optimal optical sectioning. For 3D spatial rendering, optical z-sections (z-stacks) were captured over a total depth of approximately 120 µm with a step size of ≈0.8 µm. Subsequent image acquisition, 3D volume reconstruction, and analysis were performed using the ZEN imaging software (Zeiss).

#### 2.3.4 Flow cytometry

To quantify MNP cellular association, cells were incubated following the protocols described above. Cells were subsequently recovered from the 3D cultures and resuspended in PBS at a concentration of 2.5 × 10^4^ cells per mL. Samples were analysed using the FL2 channel (575/25 nm) on a Gallios flow cytometer (Beckman Coulter). Data were processed using Kaluza software (v2.1; Beckman Coulter).

### 2.4 Magnetic hyperthermia treatment and effects on tumour cell viability

Three-dimensional cell cultures were maintained at 37 °C using a circulating water bath pump (Stryker, Medical Devices & Equipment Manufacturing Company) connected to a water-jacketed tubing system. The cultures were then exposed to an alternating magnetic field (AMF; *H* = 23.9 kA m^−1^, *f* = 228 kHz) for 30 min using a commercial AMF generator (DM3, nB Nanoscale Biomagnetics, Zaragoza, Spain).

Twenty-four hours after magnetic hyperthermia treatment, cells were released from the 3D cultures and resuspended in 1× Annexin V binding buffer (10 mM HEPES/NaOH, pH 7.4; 140 mM NaCl; 2.5 mM CaCl_2_) at a concentration of 1 × 10^6^ cells per mL. Subsequently, 5 µL of Annexin V-FITC (fluorescein isothiocyanate; *λ*_ex_ = 488 nm, *λ*_em_ = 530 nm) and 5 µL of propidium iodide (PI; *λ*_ex_ = 535 nm, *λ*_em_ = 617 nm) were added to 100 µL of the cell suspension and incubated for 15 min at room temperature in the dark (FITC Annexin V Apoptosis Detection Kit, Sigma-Aldrich). After incubation, 400 µL of 1× Annexin V binding buffer was added, and samples were analysed by flow cytometry. Data analysis was performed using Kaluza software (v2.1; Beckman Coulter).

### 2.5 Statistical analysis

Data are expressed as the mean ± SD from a minimum of two biological replicates. Statistical significance between group means was assessed using GraphPad Prism v10. One-, two-, or three-way ANOVA was applied as appropriate. A 95% confidence interval was used, and Dunnett's multiple-comparison post hoc test was performed to identify statistically significant differences.

## 3 Results and discussion

### 3.1 Characterisation of glucose-functionalised MNPs for comparative MH studies

Iron oxide magnetic nanoparticles functionalised with glucose and labelled with TAMRA were employed throughout this study. The nanoparticles correspond to the same batch as those reported in our previous work,^10^ ensuring experimental reliability and direct comparability with their extensively characterised physicochemical properties.

To enable dispersion in aqueous and biological media,^13,72^ the oleic acid-coated MNPs were transferred to water using the amphiphilic polymer PMAO, following a previously optimised phase-transfer protocol.^54^ PMAO was modified with the fluorescent dye TAMRA to facilitate *in vitro* nanoparticle tracking, allowing visualisation by confocal microscopy and quantitative assessment of cellular association 7by flow cytometry. The polymer coating was subsequently functionalised with glucose (Glc) to generate the Fe_3_O_4_@PMAO-TAMRA-Glc nanoparticles, optimised to enhance nanoparticle-cell interactions by exploiting the elevated metabolic demand of tumours^50,51^ while preserving stability under physiological conditions.^10,13,70,73^ In this context, the carbohydrate moiety can confer reduced nonspecific protein adsorption, enhanced tumour-associated cell recognition, and improved cellular uptake, potentially reducing the need for more complex multifunctional surface engineering strategies.^49,52–54^ The physicochemical and superparamagnetic behaviour of the nanoparticles were first evaluated prior to biological studies.

TEM micrographs confirmed the formation of iron oxide cores produced by thermal decomposition in organic media, yielding monodisperse spherical MNPs with an average core size of ≈11 nm (Fig. S1, SI). In addition, negligible coercivity at room temperature and high saturation magnetisation (*M*_s_ = 81 A m^2^ kg^−1^_Fe_3_O_4__) indicate superparamagnetic response and good crystallinity, in agreement with previous reports.^10,13,70^ These magnetic characteristics are consistent with earlier studies on the same batch,^10^ where AC susceptibility measurements reported dipolar interaction effects dependent on particle environment. In this context, confirming the superparamagnetic state is relevant for biomedical applications such as hyperthermia, where minimal remanence after field removal is required to avoid aggregation under physiological condition.^74^

DLS measurements were performed under physiological conditions before and after glucose functionalisation in water, cDMEM 10% FBS, and PBS (Table S1, SI). Hydrodynamic size and polydispersity index (PDI) were used as indicators of colloidal stability, as increases in both parameters are associated with nanoparticle aggregation and broadening of the size distribution. The results confirmed stable dispersions over 24 h, with hydrodynamic sizes below 100 nm and PDI ≤0.3 in biological media, consistent with previous reports for this system.^10,13,70^ This is particularly relevant, as exposure to serum-containing media induces rapid protein corona formation, which strongly influences initial nanoparticle-cell interactions.^53^ Glucose functionalisation was further confirmed by *ζ*-potential measurements, which showed a reduction in surface charge from −30 mV for bare MNPs to values below −10 mV after modification (Table S1, SI).

Magnetic heating efficiency was evaluated through specific loss power measurements of glucose-functionalised MNPs in suspension (*H* = 20 kA m^−1^, *f* = 829 kHz, [Fe] = 1 mg_Fe_ mL^−1^), yielding SLP = 104 W g_Fe_^−1^, consistent with prior reported values.^10^ Following confirmation of physicochemical stability and magnetic performance, the nanoparticles were evaluated for cellular internalisation across tumour cell lines with distinct uptake profiles.

### 3.2 Intracellular MNP accumulation in different tumour cell lines and their influence on MH efficacy

The impact of MNP internalisation on cell death following exposure to an AMF was evaluated in selected tumour cell lines using the 2D-to-3D experimental framework previously validated in our earlier studies.^10,13,70^ MIA PaCa-2 cells were included as a reference model owing to their high metabolic activity and endocytic capacity.^55–57^ In addition, our previous work demonstrated that high magnetic hyperthermia efficacy can be achieved in this cell line through optimisation of the administered nanoparticle dose.^10^

To investigate how cell line-specific features influence nanoparticle uptake patterns, HCT-116 and MCF-7 cells were studied alongside MIA PaCa-2. HCT-116 colorectal cancer cells form tight intercellular junctions and well-defined architectures^60^ that may restrict nanoparticle penetration.^58–60^ They also display chemoresistance and apoptosis-related phenotypes relevant for evaluating nanoparticle-based therapeutic strategies.^61^ In contrast, multidrug-resistant MCF-7 breast adenocarcinoma cells exhibit distinct internalisation and intracellular trafficking behaviours across nanocarriers, reflecting unique membrane dynamics.^62–65^ Their growth as compact clusters in both 2D and 3D systems is associated with reduced membrane accessibility and limited nanoparticle uptake,^27,65,66^ providing a complementary context for assessing internalisation efficiency and cellular responses.

A working range of MNP concentrations was first defined to balance nanoparticle uptake with biocompatibility, minimising basal cytotoxic effects in the absence of AMF exposure. Mitochondrial metabolic activity was assessed 24 h post-internalisation using the MTT assay at different iron concentrations of magnetic nanoparticles (0.2, 0.4, 0.6, and 1.0 mg_Fe_ mL^−1^; C0.2, C0.4, C0.6, and C1.0, respectively) (Fig. 1). The MTT assay is a well-established colourimetric method based on the reduction of tetrazolium salts by NAD(P)H-dependent oxidoreductases, reflecting overall mitochondrial activity and widely employed as an indicator of cell viability.^75–77^

**Fig. 1:**
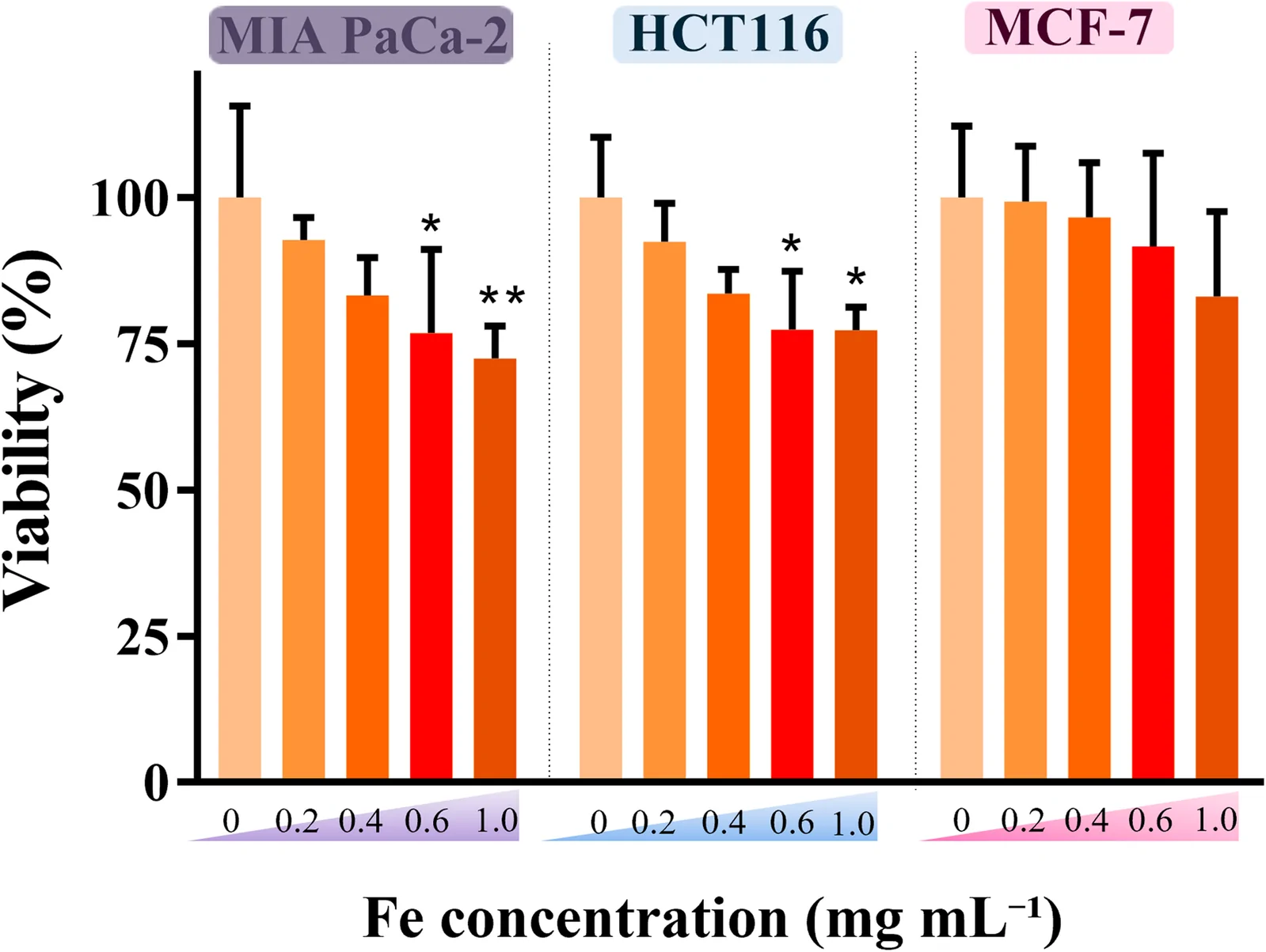
Cytotoxicity assessed by MTT 24 h after exposure of the cell lines to different MNP concentrations (mg_Fe_ mL^−1^) in a final volume of 0.25 mL. Data are presented as mean ± SD. Statistical differences were determined using two-way analysis of variance (ANOVA) followed by Dunnett's multiple comparisons test (**p < 0.01; *p < 0.05; *p* > 0.05, not significant).

The data revealed a mild, concentration-dependent decrease in metabolic activity, indicative of moderate cellular stress, in agreement with previous reports.^13,68,69^ At the lowest concentration tested (0.2 mg_Fe_ mL^−1^), both MIA PaCa-2 and HCT-116 cells maintained high viability (>90%; 93 ± 4% and 92 ± 6%, respectively). Increasing MNP concentrations resulted in a progressive reduction in metabolic activity, reaching approximately 85% at 0.4 mg_Fe_ mL^−1^ and decreasing further to around 75% at 1.0 mg_Fe_ mL^−1^ (Fig. 1 and Table S2, SI). In contrast, MCF-7 cells were less affected by MNP incubation, maintaining viability close to control levels up to 0.6 mg_Fe_ mL^−1^, with only a moderate decrease at the highest concentration (83 ± 8% at 1.0 mg_Fe_ mL^−1^). These results demonstrate differential cellular tolerance across cell lines and emphasise the importance of carefully optimised nanoparticle dosing, tailored to each cancer cell type, to preserve basal cell function, a critical prerequisite for effective MH treatment.

Considering that all selected cell lines maintained high viability at 0.4 mg_Fe_ mL^−1^ (MCF-7 >90% and HCT-116 and MIA PaCa-2 ≈83–84%, Fig. 1 and Table S2, SI), with a modest cell line-dependent variation, this concentration was selected for subsequent experiments to minimise cytotoxic interference during nanoparticle internalisation analyses. MNP cellular load was quantified after 24 h of cell-nanoparticle incubation in 2D culture. Cells were thoroughly washed to remove unbound nanoparticles and analysed by flow cytometry (Fig. 2B, D and F), providing the percentage of MNP-positive cells and the corresponding mean fluorescence intensity (MFI). The MFI reflects the relative amount of TAMRA-labelled MNPs associated with each cell, including both membrane-bound and internalised fractions.

**Fig. 2:**
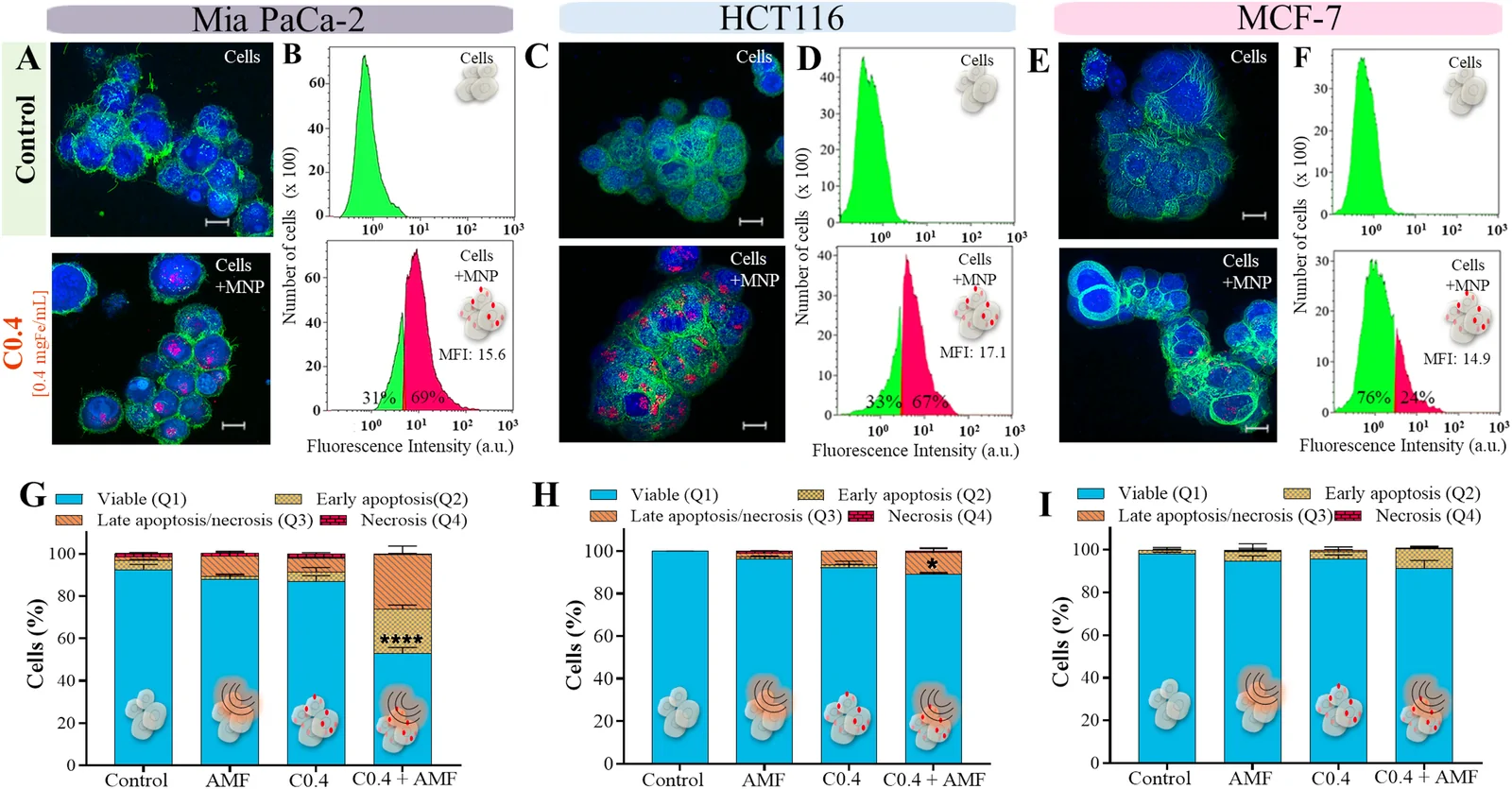
Confocal microscopy images of 3D cell cultures of MIA PaCa-2 (A), HCT-116 (C), and MCF-7 (E) incubated for 24 h with Fe_3_O_4_@PMAO-TAMRA-Glc nanoparticles at a final iron concentration of 0.4 mg_Fe_ mL^−1^ (corresponding to 100 µg mL^−1^ nanoparticles in a final volume of 0.25 mL; cDMEM), together with corresponding controls without MNPs. Cell nuclei and actin cytoskeleton were stained with DAPI (blue) and phalloidin-Alexa Fluor 488 (green), respectively. Images are shown as orthogonal projections. Scale bar: 10 µm. Images were acquired using ZEISS ZEN microscope software. (B, D and F) Flow cytometry analysis of nanoparticle uptake in the different tumour cell lines. Representative histograms from two independent experiments are shown; green indicates cells without nanoparticles and red indicates cells with internalised nanoparticles. MFI: mean fluorescence intensity. (G, H and I) Quantitative analysis of cell death induction (Annexin V/PI staining) 24 h after a single magnetic hyperthermia treatment (AMF: *H* = 23.9 kA m^−1^, *f* = 228 kHz, *t* = 30 min) in the different tumour cell lines. Data represent the mean ± SD from three independent experiments. Statistical significance relative to untreated controls was assessed using two-way ANOVA followed by Dunnett's multiple comparisons test (****p < 0.0001; *p < 0.05; *p* > 0.05 no significance). Confocal microscopy images and flow cytometry data for MIA PaCa-2 have been previously reported (reproduced from Beola *et al.*,^10^ published by the American Chemical Society, licensed under the Creative Commons Attribution 4.0 International License (CC BY 4.0), https://creativecommons.org/licenses/by/4.0) and are included here for comparative purposes. Additional image processing, including orthogonal projections and 3D reconstruction (Fig. S2, SI), was performed to provide detailed visualisation of nanoparticle distribution within cells.

To assess nanoparticle internalisation and intracellular localisation, cells were subsequently embedded in collagen-based hydrogels (Scheme 1). After gelation, samples were allowed to equilibrate for 2.5 h to ensure stable cell encapsulation in a controlled 3D environment. Samples were then fixed and imaged by confocal microscopy (Fig. 2A, C and E), enabling assessment of nanoparticle localisation within the 3D matrix and discrimination between surface-associated and internalised nanoparticles (Fig. S2 and S3, SI). Imaging was performed directly in 3D collagen gels, ensuring consistency with the magnetic hyperthermia experiments.

Cells were subsequently exposed to an AMF to evaluate the magnetic hyperthermia response (Fig. 2G–I). Confocal microscopy and flow cytometry data for MIA PaCa-2 cells reported here were acquired in parallel with HCT-116 and MCF-7 experiments using identical experimental conditions, while the latter are presented for the first time. Previously published MIA PaCa-2 data^10^ are included for comparison. Orthogonal projections (Fig. 2A, C and E) and 3D reconstructions (Fig. S3, SI) were generated for all cell lines, and multi-channel analysis, including reflection imaging (Fig. S2, SI), was performed to provide additional structural information not previously reported.

Flow cytometry analysis showed that most HCT-116 and MIA PaCa-2 cells were positive for fluorescently labelled MNPs (≈67 and 69%, respectively; Fig. 2B and D), whereas a smaller fraction of MCF-7 cells exhibited cell-associated nanoparticles (≈24%; Fig. 2F). Interestingly, the MFI of these positive subpopulations remained consistent across all three cell lines, suggesting that while the proportion of MNP-associated cells differed, the overall loading per cell was comparable. To confirm that this association reflected effective intracellular uptake, 3D cell cultures derived from these samples were examined by multi-channel confocal microscopy. High-resolution imaging at the equatorial plane and corresponding z-projections (Fig. 2A, C, E and S2, S3, SI) confirmed internalisation of TAMRA-labelled MNPs, predominantly distributed within the cytoplasmic region between the DAPI-stained nuclei and the F-actin cortical cytoskeleton. This was further supported by confocal reflection mode imaging (Fig. S2-I, SI), which provided a label-independent map of the MNP cores. The backscattered signal showed clear spatial co-localisation with TAMRA fluorescence in dual-channel overlays (Fig. S2-III, SI). Collectively, these results confirm successful intracellular uptake of MNPs across all cell lines, while revealing differences in the fraction of MNP-positive cells and their spatial distribution patterns.

Overall, while MIA PaCa-2 and HCT-116 cells exhibited comparable uptake at the population level, MCF-7 cells showed reduced internalisation, consistent with the limited changes in metabolic activity observed in the MTT assay (Fig. 1). This behaviour is in line with previous reports indicating that the growth characteristics of MCF-7 cells in culture, including their tendency to form compact clusters that limit surface accessibility during the initial incubation, can influence the efficiency of nanoparticle uptake among different nanomaterial systems.^27,63,78^ Nevertheless, variability in nanoparticle uptake among cell lines is recognised as a multifactorial process, influenced not only by nanoparticle physicochemical properties and experimental conditions, but also by cell-specific features such as growth behaviour, organisation, and metabolic phenotype.^24,79–84^ In this context, glucose transporter 1 (GLUT1, SLC2A1), a key mediator of glucose uptake and marker of glycolytic activity, is differentially expressed across tumour cell lines.^50,85,86^ While breast cancer cells such as MCF-7 exhibit a more moderate glycolytic profile in 2D culture,^86,87^ colorectal (HCT-116) and pancreatic (MIA PaCa-2) cells display higher GLUT1-associated glycolytic activity.^88–90^ These differences in metabolic state and growth behaviour may therefore contribute to the reduced nanoparticle association observed in MCF-7 cells relative to HCT-116 and MIA PaCa-2.

To evaluate the effect of nanoparticle uptake on the MH treatment response, 3D cultures were exposed to an alternating magnetic field (AMF; *H* = 23.9 kA m^−1^, *f* = 228 kHz, *t* = 30 min), and the resulting biological effects were assessed after 24 h by flow cytometry (Fig. 2G–I). Annexin V (AnnV) and propidium iodide (PI) staining allowed discrimination between viable cells and distinct modes of cell death.^91,92^ Early apoptosis is characterised by externalisation of phosphatidylserine to the outer layer of the plasma membrane, while membrane integrity is maintained, allowing Annexin V to bind PS (AnnV^+^/PI^−^). As apoptosis progresses, membrane permeabilisation enables PI entry and nucleic acid binding, yielding late apoptotic (AnnV^+^/PI^+^) and necrotic signals (AnnV^−^/PI^+^).^93^ This combined analysis provides mechanistic insight into the progressive cell death pathways induced by MH and allows direct comparison of cytotoxic responses across tumour cell lines with different levels of MNP uptake.

Although HCT-116 cells exhibited fractions of MNP-positive cells and MFI values comparable to those of MIA PaCa-2 in parallel experiments, their cytotoxic response to magnetic hyperthermia was substantially lower. MIA PaCa-2 cells previously showed a pronounced treatment effect under these conditions (≈53 ± 3%),^10^ reflecting the total fraction of cells across all stages of cell death (Fig. 2G), whereas HCT-116 cells responded only modestly (≈11 ± 1%, Fig. 2H). This indicates that comparable nanoparticle internalisation does not necessarily translate into equivalent therapeutic efficacy. MCF-7 cells, on the other hand, exhibited a similarly weak cytotoxic response following MH treatment (≈9 ± 4%, Fig. 2I), consistent with limited nanoparticle-cell interaction (Fig. 2E, F and S2, S3, SI). Collectively, these results highlight that MH efficacy cannot be predicted solely from overall MNP internalisation, and that additional cell-specific factors modulate treatment response. In particular, the limited MH efficacy observed in HCT-116 and MCF-7 cells under conditions effective in MIA PaCa-2 motivated the exploration of strategies to modulate nanoparticle-cell interactions and assess their impact on MH responses in these less responsive tumour cell lines.

On this basis, two complementary strategies were evaluated to enhance nanoparticle-cell interactions in tumour models with low treatment sensitivity. Increasing the MNP concentration in the culture medium is a common approach to enhance MH-induced cytotoxicity,^10,13,34^ but it can be limited by potential dose-dependent toxicological effects.^94^ Therefore, we assessed in parallel the use of an external static magnetic field (MF) gradient, promoting nanoparticle accumulation at the cell surface and internalisation without raising the administered dose.^36,37,95,96^

### 3.3 Comparative analysis of two strategies to modulate MNP-cell interactions

To assess these strategies, MCF-7 and HCT-116 cells cultured as 2D monolayers were incubated with MNPs at two concentrations (0.4 and 1.0 mg_Fe_ mL^−1^) in the presence or absence of a static MF gradient generated by a Nd–Fe–B permanent magnet (≈350 mT) positioned under the culture plates (Scheme 1).

The lower concentration (0.4 mg_Fe_ mL^−1^) was selected based on previous observations of MH-induced cell death in MIA PaCa-2 cells (Fig. 2G). MF exposure promotes local redistribution and accumulation of MNPs at the cell surface without altering the bulk nanoparticle concentration in the culture medium.^36,38,97^ The higher concentration (1.0 mg_Fe_ mL^−1^) was included to evaluate whether increasing the global nanoparticle dose enhances cellular association and MH efficiency, both in the absence and presence of the MF.

To visualise the early effects of the static MF on MNP behaviour, optical microscopy images were acquired five minutes after MF application in the 0.4 mg_Fe_ mL^−1^ + MF condition. These images revealed rapid MNP alignment in response to the applied field, confirming that the MF gradient was sufficient to magnetically manipulate the administered MNPs at early time points (Fig. 3). This behaviour is consistent with field-induced nanoparticle sedimentation towards the cell interface, increasing local particle concentration and promoting cell-particle interactions, in line with previous reports on magnetic nanoparticle uptake *in vitro*.^39,98–100^

**Fig. 3:**
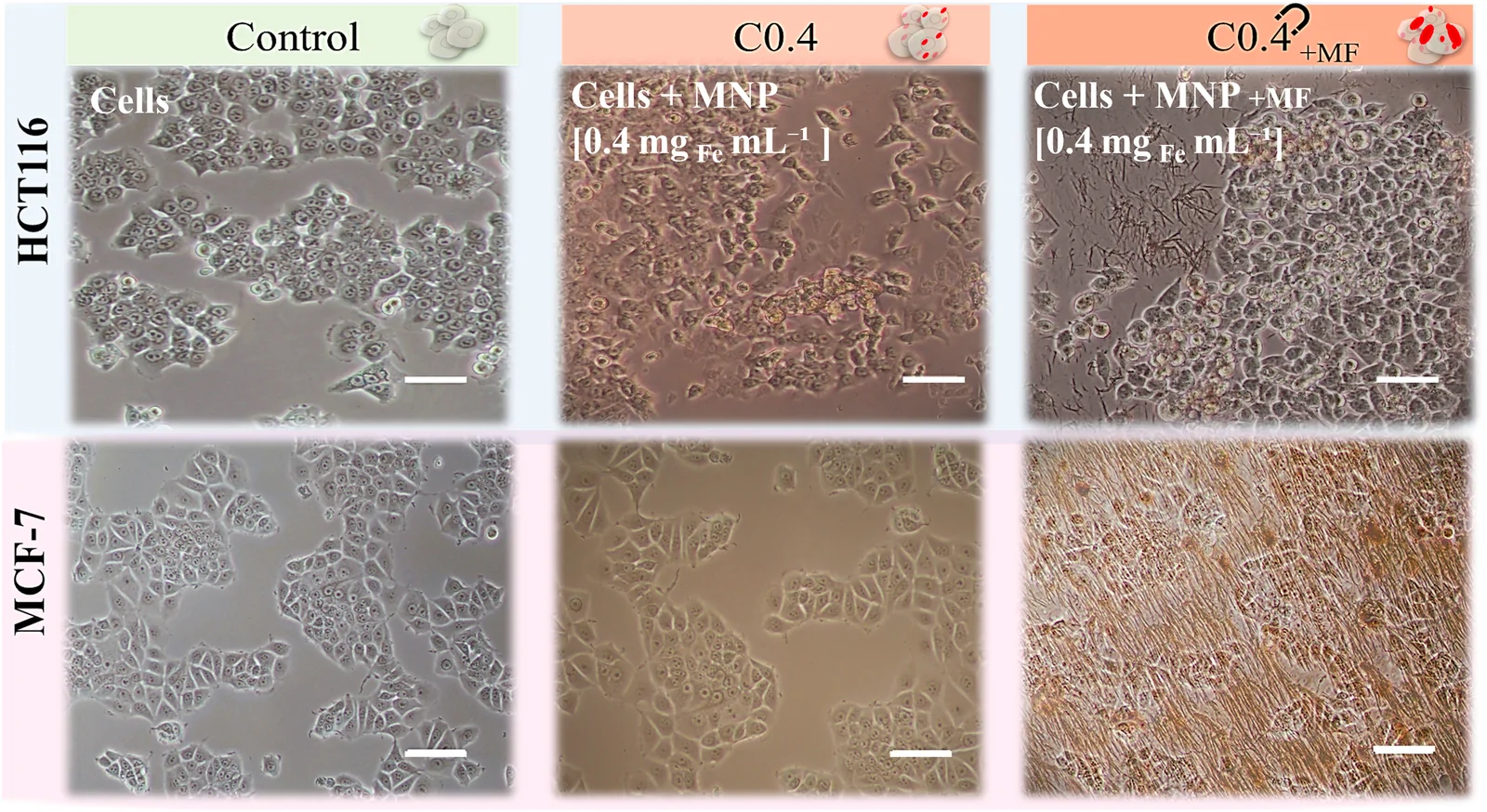
Brightfield images of monolayer-cultured cells acquired 5 min after incubation with Fe_3_O_4_@PMAO-TAMRA-Glc (0.4 mg_Fe_ mL^−1^) in the presence or absence of a static magnetic field (MF) gradient (≈350 mT). Scale bars: 20 µm.

After 24 h of incubating the cells with MNPs, the MF was removed where applicable, and the MNP-containing medium was discarded in all cases. Cells were then washed extensively to remove non-associated MNPs prior to detachment, and flow cytometry analysis to quantify cell-associated nanoparticles (Fig. 4). The analysis revealed that application of a static MF gradient at 0.4 mg_Fe_ mL^−1^ significantly increased the fraction of MNP-positive cells in both lines (HCT-116: 67% to 86%; MCF-7: 24% to 93%), approaching the levels observed at 1.0 mg_Fe_ mL^−1^ without MF (85% and 94%, respectively) (Fig. 4A and B). In all cases, the MFI, indicative of MNPs load per cell, remained unchanged (Fig. 4A). Notably, combining a higher MNP concentration with MF exposure (C1.0_+MF_) did not further increase nanoparticle cellular association and MFI (Fig. S4, SI), suggesting that this process reaches saturation in both cell lines.

**Fig. 4:**
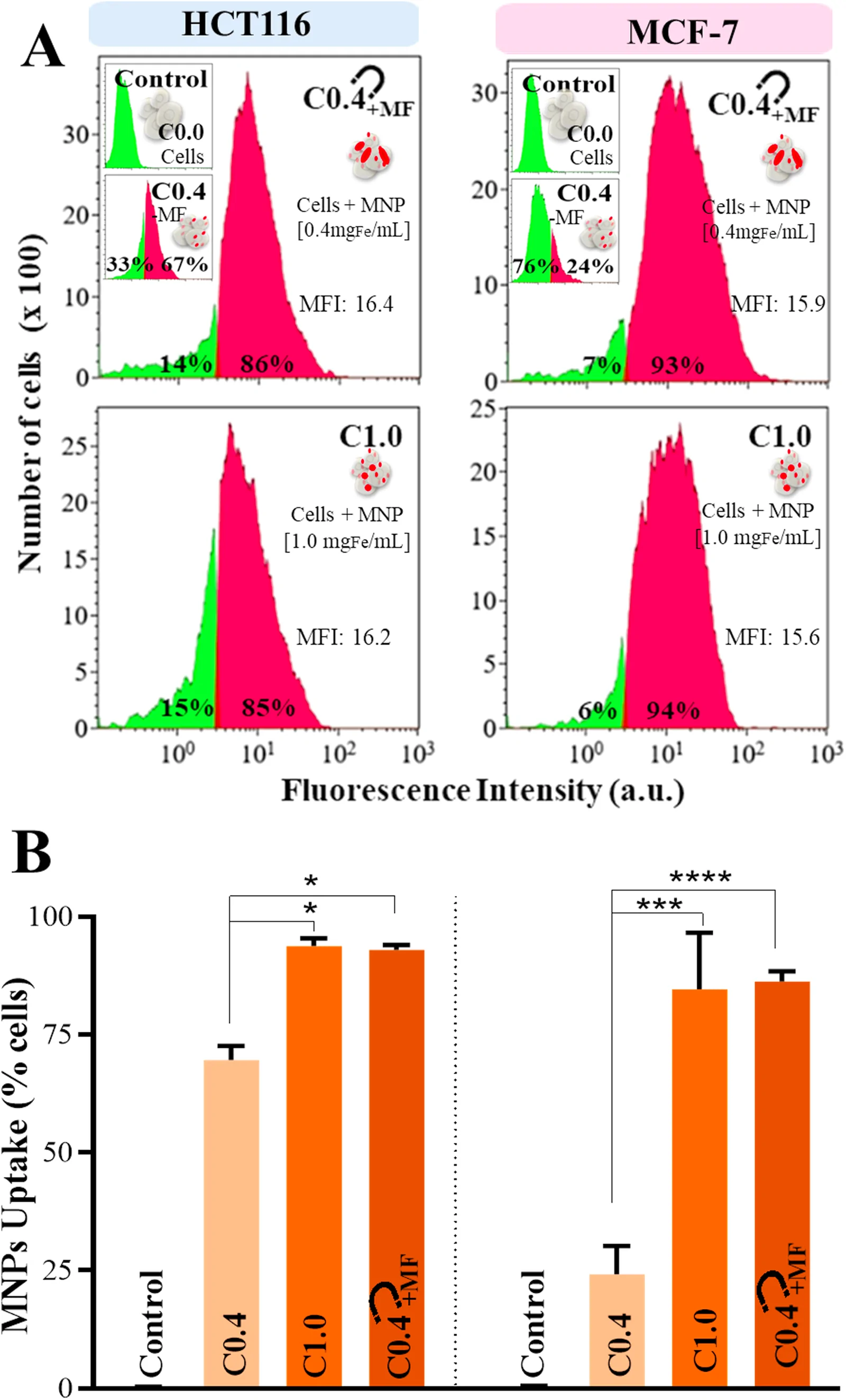
Flow cytometry analysis of MNP-associated MCF-7 and HCT-116 cells 24 h post-incubation under different experimental conditions. (A) Representative flow cytometry histograms showing the distribution of fluorescence intensity for cells incubated with MNPs at 0.4 mg_Fe_ mL^−1^ in the presence of a static magnetic field (C0.4_+MF_; MF ≈350 mT) and at 1.0 mg_Fe_ mL^−1^ in the absence of MF (C1.0). For the C0.4_+MF_ condition, the inset shows the corresponding histogram obtained at the same concentration (0.4 mg_Fe_ mL^−1^) without MF (C0.4), used as an internal reference. Histograms are representative of two independent experiments. MFI: mean fluorescence intensity. (B) Quantification of the percentage of MNP-positive cells determined by flow cytometry, expressed as mean ± SD from two independent experiments. Cells incubated in the absence of MNPs (0.0 mg_Fe_ mL^−1^ = Control) were used as a negative control to establish the fluorescence threshold. Statistical differences were assessed by two-way analysis of variance (ANOVA) followed by Dunnett's multiple comparisons test (*p < 0.05; **p < 0.01; ***p < 0.001; ****p < 0.0001; n.s., not significant).

The effect of the MF can be attributed to two complementary mechanisms. First, MF gradients promote rapid sedimentation and redistribution of MNPs toward the cell surface, increasing the fraction of the administered dose that is effectively available to cells during early incubation times.^36,38,97^ Second, the resulting local increase in local particle density at the cell-medium interface may facilitate engagement of cellular uptake pathways, including clathrin- and caveolin-mediated endocytosis, which have previously been reported to contribute to magnetically assisted nanoparticle internalisation.^37,96^

While both strategies, increasing MNP concentration and applying an MF gradient, enhance nanoparticle availability at the cell surface and promote cellular association, they operate through distinct mechanisms. Increasing MNP concentration raises particle burden in the culture environment and correlates with reduced metabolic activity at higher doses (≈23% reduction in HCT116 and ≈17% in MCF-7 at 1.0 mg_Fe_ mL^−1^; Fig. 1). This differential behaviour between cell lines is consistent with their distinct biological characteristics.^27,58–61,65,66^ By contrast, MF-assisted conditions (0.4 mg_Fe_ mL^−1^) achieved comparable percentages of MNP-positive cells, as confirmed by flow cytometry (Fig. 4), without proportionally increasing the global nanoparticle dose. These observations suggest that magnetic guidance improves MNP-cell interaction efficiency while limiting the need for elevated nanoparticle concentrations in the surrounding medium, which may favour more controlled cellular responses by reducing stress associated with excessive MNPs exposure.^23,101–103^

Importantly, the static MF applied (≈350 mT) is considered moderate and is commonly used in magnetofection and magnetic targeting.^41,95^ Under these conditions, the field primarily acts on the nanoparticles rather than directly on the cells and has not been widely reported to induce structural or genetic alterations.^36,39–41,95,96^ Moreover, the absence of measurable cytotoxic effects of the MF gradient on cells in the absence of nanoparticles (Control_+MF_) suggests that static MF exposure does not introduce additional toxicity beyond that associated with MNP exposure (C1.0_+MF_) (Fig. S5, SI). Cellular effects have generally been reported only under more extreme conditions, such as dynamic fields, prolonged exposures, or substantially higher intensities,^37^ which is consistent with the use of MF gradients for enhancing MNP-cell interactions under the conditions applied in this study.

### 3.4 Effect of MNP-cell interaction modulation on magnetic hyperthermia response

Building on these findings, we next examined whether the incubation conditions that enhanced MNP-cell interactions in 2D cultures could influence MH responses in a 3D collagen-based system, which provides a more physiologically relevant environment (Scheme 1). For that, cells were pre-incubated under monolayer conditions for 24 h with 0.4 mg_Fe_ mL^−1^ under a static MF gradient (C0.4_+MF_) or 1.0 mg_Fe_ mL^−1^ without MF (C1.0), then embedded in collagen and exposed to mild MH treatment (*H* = 23.9 kA m^−1^, *f* = 228 kHz, *t* = 30 min), generating the C0.4_+MF_ + AMF and C1.0 + AMF conditions. MH-induced cytotoxicity was assessed by flow cytometry 24 h post-AMF (Fig. 5). Controls included 3D cultures prepared from cells without nanoparticles, either exposed (Control_+MF_; Fig. S5, SI) or not to MF (Control; Fig. 5) and subsequent AMF (Control + AMF and Control_+MF_ + AMF; Fig. 5 and S5, SI respectively), as well as additional reference conditions (C0.4, C0.4 + AMF and C1.0_+MF_; Fig. S5, SI) to provide a complete framework for comparison.

**Fig. 5:**
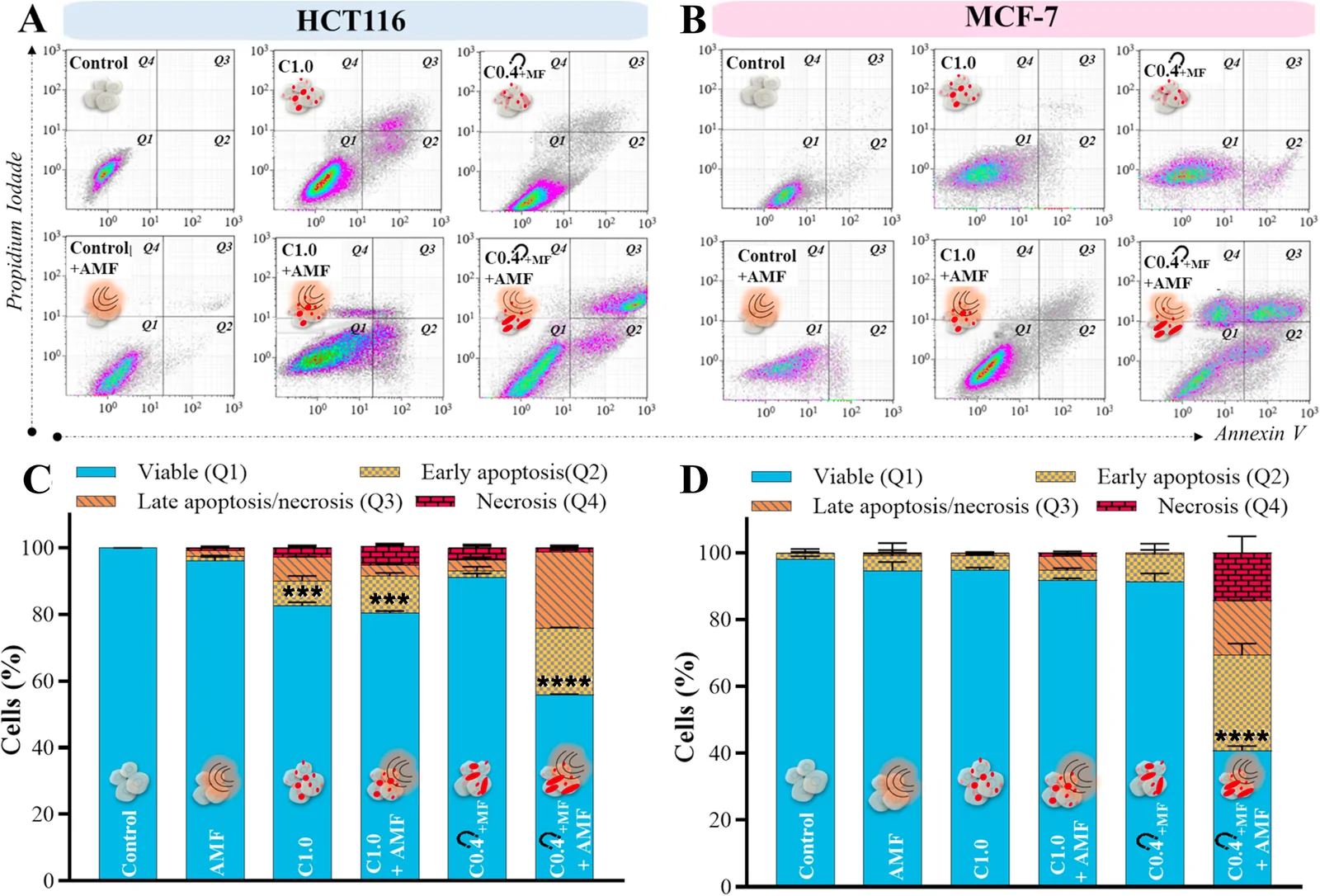
Magnetic hyperthermia-induced cell death in different tumour cell types (Annexin V and PI staining). (A and B) Representative density plots 24 h post-MH treatment (two independent experiments); quadrants indicate viable (Q1: Annexin V^−^/PI^−^), early apoptotic (Q2: Annexin V^+^/PI^−^), late apoptotic/necrotic (Q3: Annexin V^+^/PI^+^), and necrotic (Q4: Annexin V^−^/PI^+^) cells. (C and D) Summary of flow cytometry data (mean ± SD, *n* = 2). Statistical significance of differences in viable cells was determined relative to untreated control cells without nanoparticles using one-way ANOVA with Dunnett's multiple comparisons test.

As shown in Fig. 5, MH treatment induced markedly different cytotoxic responses depending on the strategy used to promote nanoparticle uptake. Cells pre-incubated under a static MF gradient (C0.4_+MF_) exhibited significantly higher MH-induced cell death than those incubated with a higher MNP concentration without MF (C1.0) or with a lower concentration without MF (C0.4; Fig. S5, SI). This trend was consistent across both cell lines, with the magnitude of the response reflecting cell type differences, further emphasising the significantly enhanced effectiveness of MF-assisted delivery in promoting MH-induced tumour cell cytotoxicity. In MCF-7 cells, C0.4_+MF_ + AMF induced ≈61% cell death (Fig. 5B and D), compared to ≈9% and ≈8% for C0.4 + AMF (Fig. S5B, SI) and C1.0 + AMF (Fig. 5B and D), respectively. In HCT-116 cells, C0.4_+MF_ + AMF resulted in ≈44% cell death (Fig. 5A and C), *versus* ≈11% and ≈20% for C0.4 + AMF (Fig. S5A, SI) and C1.0 + AMF (Fig. 5A and C). Notably, 1.0 mg_Fe_ mL^−1^ (C1.0) without AMF caused moderate cytotoxicity in HCT-116, reflecting higher sensitivity to nanoparticles alone (Fig. 5A, C and Table S3, SI). Our data show that neither the static MF gradient nor the AMF induced detectable cytotoxic effects on cells incubated in the absence of nanoparticles (Control_+MF,_ Control_+MF_ + AMF; Fig. S5, SI and Control + AMF; Fig. 5 respectively), as no significant changes in cell viability or baseline responses (Control cells) were observed under either condition. Furthermore, three-way ANOVA revealed significant interactions between incubation strategy and MH treatment (*p* <0.05–<0.0001, Table S5, SI), supporting the statistical robustness of the observed differences.

These results demonstrate that similar levels of cellular nanoparticle association can yield markedly different magnetic hyperthermia outcomes in 3D cultures. Notably, the cellular MNP cargo was largely preserved during therapy, as the experimental setup was designed to minimise the contribution of non-associated nanoparticles.^10,13,70^ The combination of stringent washing protocols and the subsequent 3D encapsulation process effectively removed weakly adsorbed MNPs from the cell surface. This is further supported by single-plane confocal micrographs (Fig. S2, SI), which consistently show TAMRA signal confined within the intracellular volume rather than at the cell periphery. Moreover, the correlation between intracellular fluorescence intensity and cytotoxic response across cell lines suggests that the observed effects are primarily driven by internalised nanoparticles rather than membrane-associated fractions, although further investigation is warranted.

While enhanced cytotoxicity manifests upon AMF exposure, the data do not reveal a specific mechanism linking MF-assisted incubation to hyperthermia, suggesting that incubation conditions prime the nanoparticle-cell system for a functionally relevant response. Contributing factors may include differences in nanoparticle distribution, aggregation, or interaction patterns, as well as cell-specific responses to higher MNP concentrations.^35,96,104^ AMF-induced dynamic nanoparticle rearrangements, such as chain formation, may also influence local heat generation,^105^ although direct correlations with biological effects are not fully established.

In particular, MH efficacy in 3D tumour-like models reflects a complex interplay beyond nanoparticle dose alone, emphasising the critical role of the manner in which MNP-cell interactions are established prior to treatment. These results highlight the role of MF-assisted delivery within therapeutic outcomes, providing a basis for future mechanistic and applied studies.

## 4 Conclusions

Achieving effective antitumoral magnetic hyperthermia remains challenging due to the complex, cell-dependent nature of nanoparticle-cell interactions. Comparison of MCF-7 breast and HCT-116 colon carcinoma cells with the MIA PaCa-2 pancreatic reference model demonstrated that comparable levels of nanoparticle cellular association do not necessarily translate into similar MH responses. This indicates that nanoparticle loading alone is insufficient to predict therapeutic response, and that additional cell-specific biological factors critically modulate MH sensitivity.

Distinct limitations were identified in each tumour model. MCF-7 cells exhibited reduced MH efficacy at low nanoparticle concentrations due to limited cellular interaction with the nanoparticles, whereas HCT-116 cells displayed levels of cell-associated nanoparticles comparable to the reference model but comparatively lower MH-induced cytotoxicity. To address these limitations, two nanoparticle-delivery enhancement strategies were evaluated: increasing nanoparticle concentration and applying a static magnetic field gradient during incubation. Among them, MF-assisted incubation consistently improved MH-induced cytotoxicity, including under conditions where equivalent nanoparticle concentrations alone produced limited therapeutic effects.

Importantly, these findings indicate that MH efficacy depends not only on nanoparticle dose, but also on the manner in which nanoparticles interact with cells under defined conditions. In this context, static MF guidance provides an externally controllable strategy capable of enhancing cellular nanoparticle accumulation without increasing overall nanoparticle dose. This approach complements ligand-based targeting strategies, including carbohydrate functionalisation, by reducing dependence on receptor expression and intrinsic uptake variability, thereby promoting more consistent intracellular nanoparticle accumulation across heterogeneous cellular phenotypes. In contrast to dose-escalation strategies, which may increase nonspecific nanoparticle-associated stress responses, the combination of MF guidance with biochemical targeting offers a more versatile framework to optimise magnetothermal performance. Overall, these results establish MF-guided incubation as an effective strategy to enhance MH efficacy through improved control of nanoparticle transport and cellular interaction dynamics, highlighting the need to move beyond dose-centric paradigms and to integrate biophysical guidance mechanisms with cell-specific targeting strategies in the rational design of magnetothermal therapies.

Although the present results demonstrate the potential of MF-assisted nanoparticle delivery under controlled *in vitro* conditions, the biological and biophysical mechanisms underlying MF-associated enhancement effects beyond nanoparticle association/internalisation remain incompletely understood and warrant further investigation. Future studies should therefore focus on elucidating these mechanisms and evaluating the applicability of this strategy in more advanced and physiologically relevant preclinical models.

## Author contributions

L. B.: conceptualization, investigation, data curation, formal analysis, writing – original draft, writing – review & editing. L. G.: conceptualization, methodology, data curation, writing – review & editing. R. M. F.: methodology, writing – review & editing. J. M. F.: resources, writing – review & editing. L. A.: conceptualization, methodology, supervision, writing – review & editing. V. G.: conceptualization, funding acquisition, writing – review & editing.

## Conflicts of interest

There are no conflicts to declare.

## Supplementary Material

NA-008-D6NA00172F-s001

## Data Availability

The data supporting this article have been included as part of the supplementary information (SI). Supplementary information: transmission electron microscopy characterisation, particle size distribution and field-dependent magnetisation measurements of Fe_3_O_4_@PMAO-TAMRA-Glc nanoparticles (Fig. S1); physicochemical properties of MNPs, including hydrodynamic diameter and ζ-potential (Table S1); cell viability in tumour lines following MNP exposure (Table S2); multi-modal confocal microscopy analysis of MNP uptake within a 3D cellular environment (Fig. S2); three-dimensional confocal microscopy reconstruction of 3D cultures (Fig. S3); MNP uptake analysed by flow cytometry under a static magnetic field (Fig. S4); induction of cell death assessed by Annexin V/PI staining 24 h post-treatment (Fig. S5); summary of Annexin V/PI cell death analysis (Table S3); and statistical evaluation of cell viability differences following AMF exposure (Table S4). See DOI: https://doi.org/10.1039/d6na00172f.
