# Supplementary material for: Magnetic field-guided enhancement of nanoparticle uptake and hyperthermia efficacy in 3D tumour models
Source: Nanoscale Adv. 2026 Aug 20. Online ahead of print. doi: 10.1039/d6na00172f (PMC13523097; doi:10.1039/d6na00172f)
Supplement: Supplementary file 1 [file NA-OLF-D6NA00172F-s001.pdf]

Supplementary Information (SI) for Nanoscale Advances.
This journal is © The Royal Society of Chemistry 2026

Supplementary Information (SI)

Magnetic field–guided enhancement of nanoparticle uptake and hyperthermia efficacy in 3D tumour models

*Lilianne Beola *[a,b], Lucía Gutiérrez [a,b], Raluca M. Fratila [a,b], , Jesús M. de la Fuente [a,b], Laura Asín †[a,b], Valeria Grazú *[a,b].*

[a] Instituto de Nanociencia y Materiales de Aragón, INMA (CSIC-Universidad de Zaragoza), C/ Pedro Cerbuna 12, 50009, Zaragoza, Spain.

[b] Centro de Investigación Biomédica en Red de Bioingeniería, Biomateriales y Nanomedicina (CIBER-BBN), 50018 Zaragoza, Spain.

† Current address: Department of BioChemistry and Molecular and Cellular Biology / Institute for Biocomputation and Physics of Complex Systems, University of Zaragoza, 50018 Zaragoza, Spain.

Corresponding authors: Lilianne Beola (lbeol@unizar.es) and Valeria Grazú (vgrazu@csic.es).

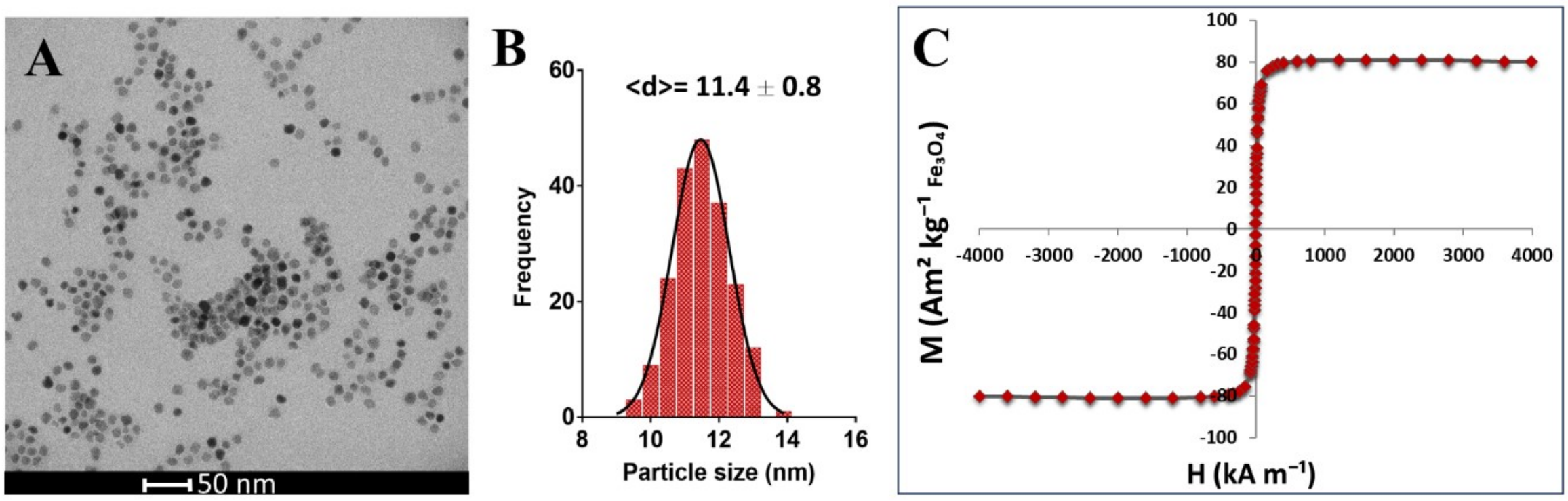

Figure S1. Characterisation of Fe_3O_4@PMAO-TAMRA-Glucose nanoparticles. (A) *Transmission electron microscopy (TEM) image showing nanoparticle morphology and dispersion.* ***(B)*** *Particle size distribution histogram obtained from TEM image analysis.* ***(C)*** *Field dependent magnetization measurements obtained showing a superparamagnetic behavior at 300 K.*

Table S1. Hydrodynamic diameter and ζ-potential of functionalised magnetic nanoparticles. *Hydrodynamic size (intensity-based) and zeta potential (ζ) of MNPs coated with PMAO and TAMRA (MNPs@PMAO-TAMRA), measured in water, and after glucose functionalisation (MNPs@PMAO-TAMRA@Glucose), measured in phosphate-buffered saline (PBS, pH 7.0) and following 24 h incubation in complete DMEM (cDMEM).*

Sample	Suspension media	Hydrodynamic size (nm)	Polydispersity index (PDI)	ζ potential (mV)
Fe_3O_4@PMAO-TAMRA	Water	69	0.5	-30
Fe_3O_4@PMAO- TAMRA@Glc	PBS	99	0.6	-9.28
Fe_3O_4@PMAO-TAMRA@Glc	cDMEM	72	0.3	-7.81

Table S2. Cell viability after MNP exposure in different tumour cell lines. *Cell viability assessed by MTT assay following 24 h incubation with increasing concentrations of magnetic nanoparticles (MNPs@PMAO-TAMRA@Glucose). Data are presented as mean ± standard deviation (n = 3).*

Cell lines	0.0 mg_{Fe} mL^{-1}	0.2 mg_{Fe} mL^{-1}	0.4 mg_{Fe} mL^{-1}	0.6 mg_{Fe} mL^{-1}	1.0 mg_{Fe} mL^{-1}
Mia Paca-2	100 ± 15	93 ± 4	83 ± 6	77 ± 14	72 ± 6
HCT116	100 ± 10	92 ± 6	84 ± 4	77 ± 10	77 ± 4
MCF-7	100 ± 12	99 ± 7	97 ± 9	92 ± 15	83 ± 8

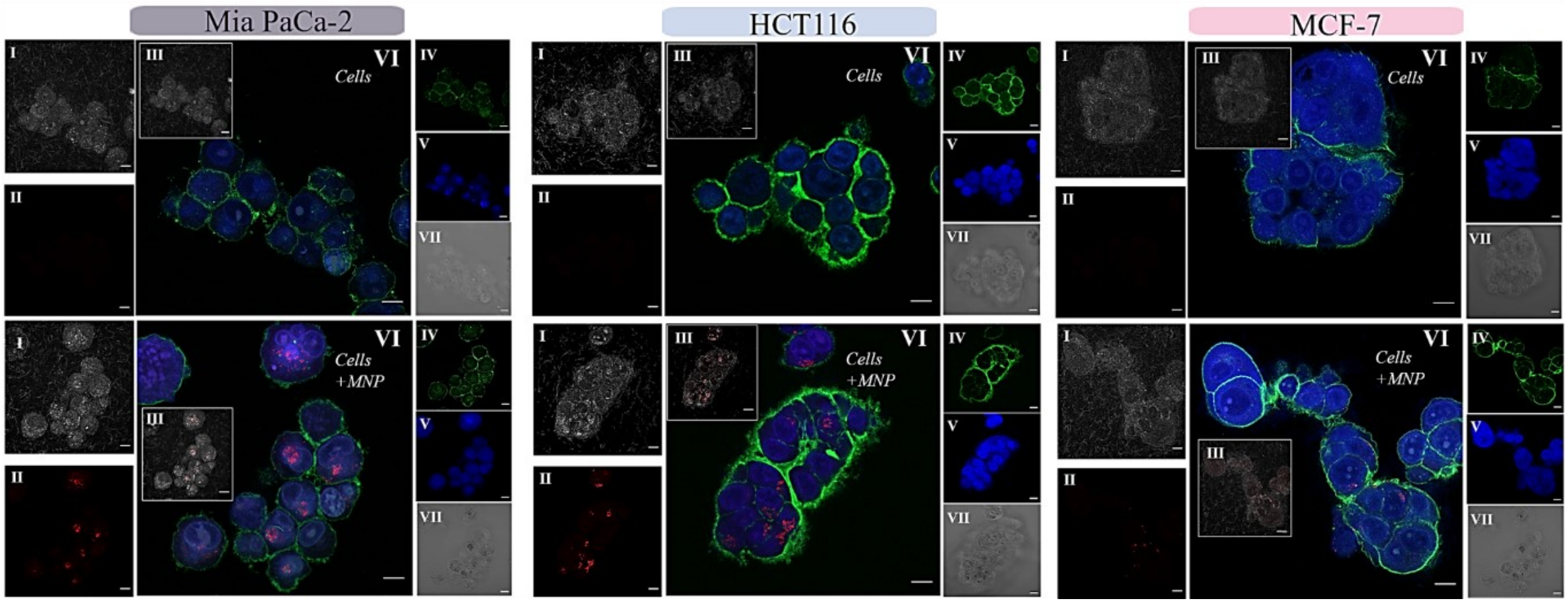

Figure S2. *Multi-modal confocal microscopy analysis of MNP uptake within a 3D cellular environment. (I) Confocal reflection mode image (greyscale) revealing the physical distribution of MNP cores via light scattering. (II) Fluorescence signal from TAMRA-labelled MNPs (red). (III) Overlay of the TAMRA fluorescence and the reflection signal, demonstrating precise spatial co-localisation of the fluorophore and the nanoparticle core. (IV) Cell nuclei stained with DAPI (blue). (V) F-actin cytoskeleton visualised with Phalloidin-Alexa Fluor 488 (green). (VI) Composite merged image showing the spatial relationship between nuclei (blue), cytoskeleton (green), and internalised MNPs (red). (VII) Phase-contrast micrograph illustrating the 3D cell colony morphology. Confocal images of MIA PaCa-2 have been previously reported (Reproduced from ACS Appl. Mater. Interfaces 2021, 13, 11, 12982–12996, © 2021 Beola et al., published by the American Chemical Society, and licensed under the Creative Commons Attribution 4.0 International License, CC BY 4.0, https://creativecommons.org/licenses/by/4.0). These data are included for comparative and contextual purposes and form the basis for the strategy-driven analysis presented in this work. Scale bars: 10 µm.*

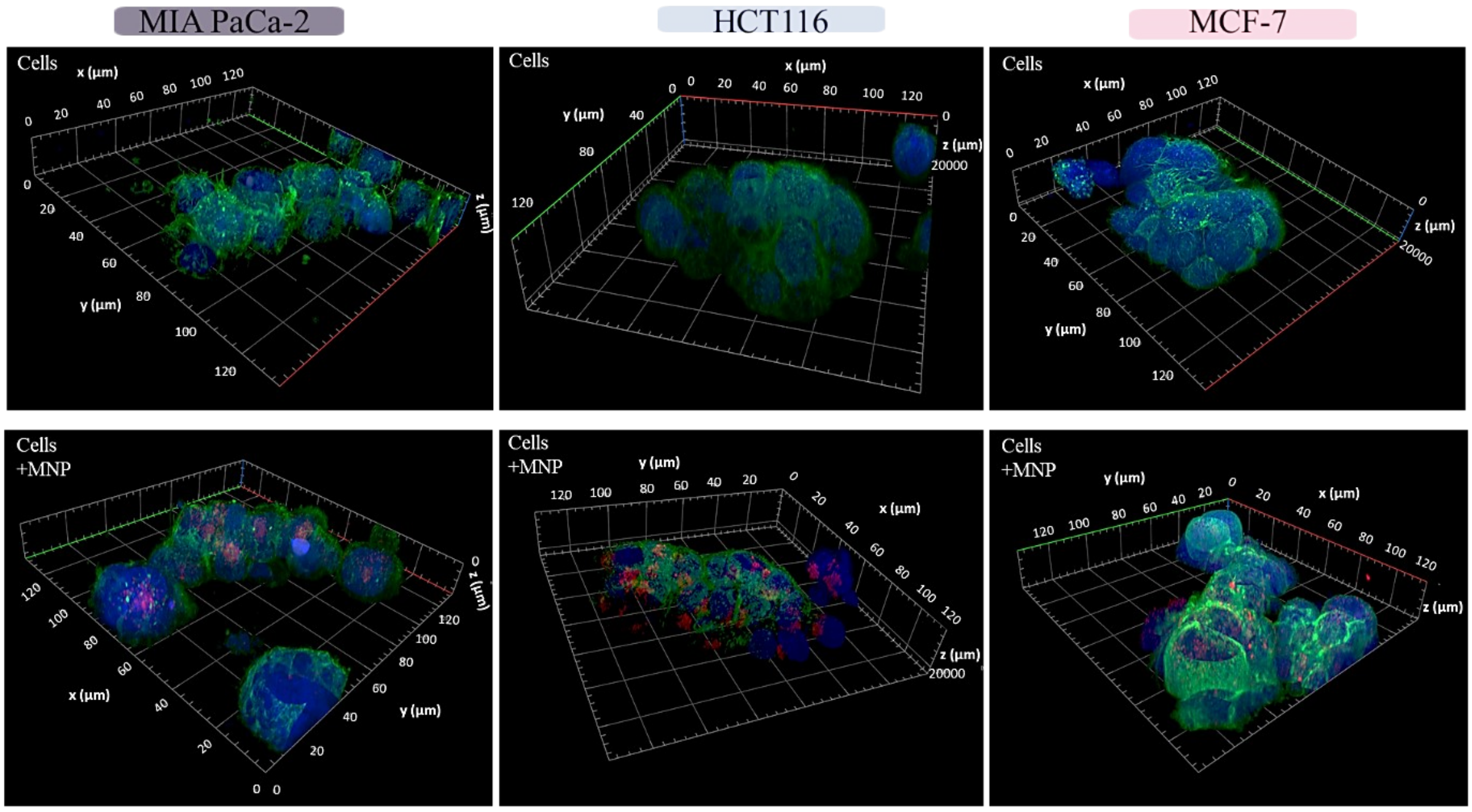

Figure S3. Three-dimensional projections of confocal microscopy images of 3D cell cultures *of MIA PaCa-2, HCT-116 and MCF-7 following 24 h incubation with 100 µg mL^{-1} Fe_3O_4@PMAO-TAMRA-Glc nanoparticles (red fluorescence) in a final volume of 0.25 mL (cDMEM; C0.4), together with corresponding controls without MNPs. Cell nuclei and the actin cytoskeleton were stained with DAPI (blue) and phalloidin–Alexa Fluor 488 (green), respectively. Confocal images of MIA PaCa-2 have been previously reported (Reproduced from ACS Appl. Mater. Interfaces 2021, 13, 11, 12982–12996, © 2021 Beola et al., published by the American Chemical Society, and licensed under the Creative Commons Attribution 4.0 International License, CC BY 4.0, https://creativecommons.org/licenses/by/4.0). These data are included for comparative and contextual purposes and form the basis for the strategy-driven analysis presented in this work.*

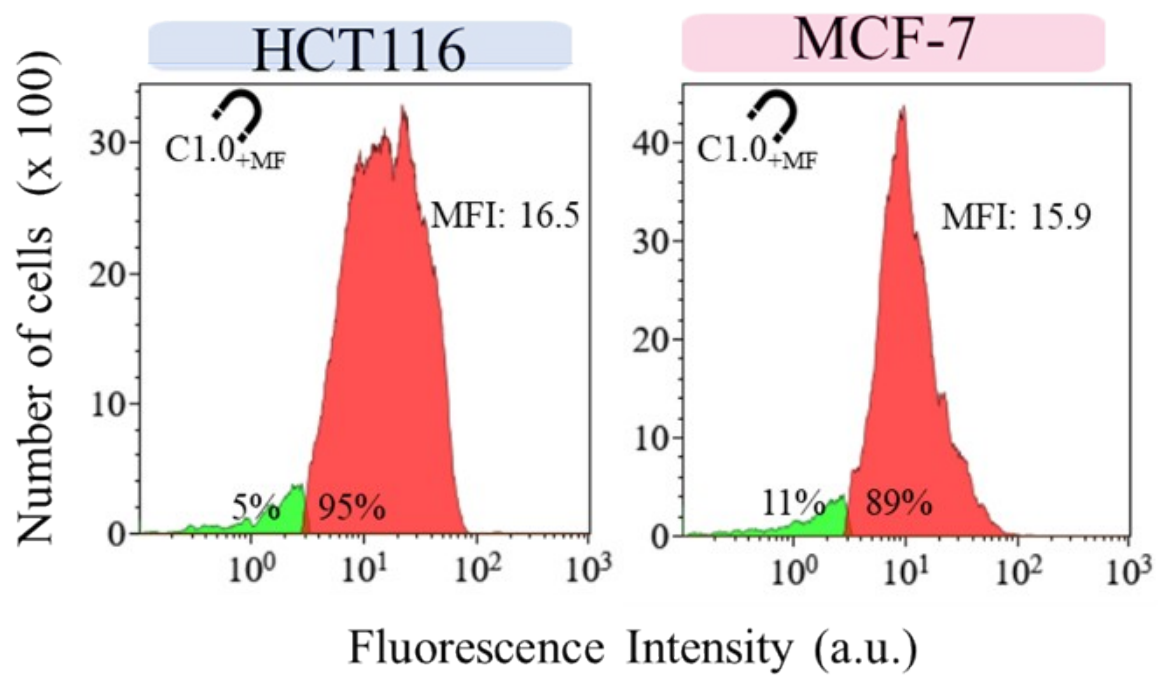

Figure S4. Flow cytometry analysis of MNP uptake by tumour cells following exposure to a static magnetic field gradient. *MCF-7 and HCT116 cells were incubated for 24 h with 250 µg Fe of magnetic nanoparticles in a final volume (cDMEM) of 0.25 mL (C 1.0) under a static MF gradient (≈350 mT) generated by Nd-Fe-B permanent magnets positioned beneath the culture plates (C1.0$_{+MF}$ condition). Following incubation, the magnets were removed and cells were detached and thoroughly washed to remove unbound MNPs. Uptake was analysed by flow cytometry. Histograms show the proportions of MNP-negative (green) and MNP-positive (red) cells.*

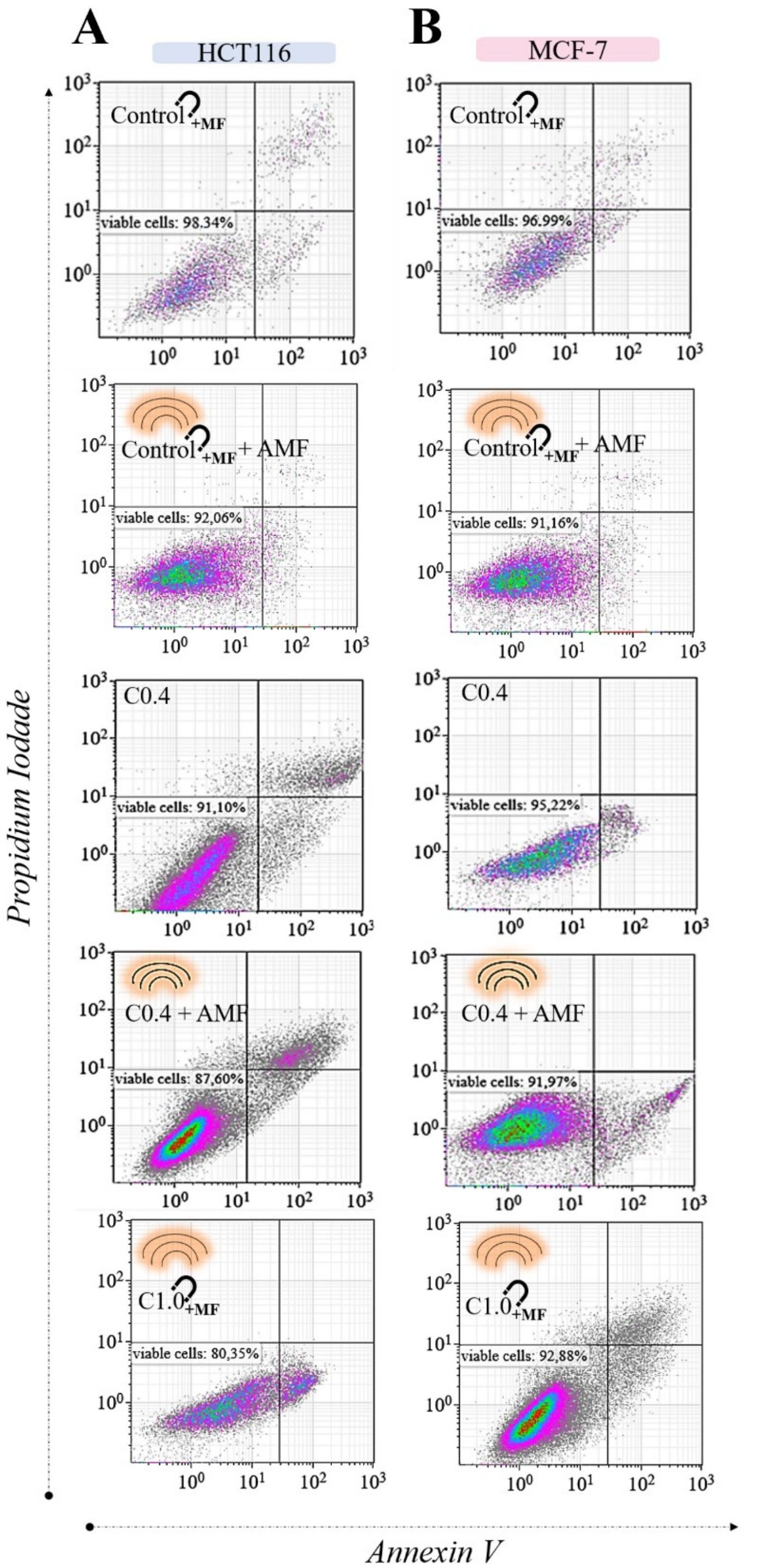

Figure S5. Induction of cell death in tumour cells assessed by flow cytometry (Annexin V/PI staining) 24 h after magnetic hyperthermia treatment. *Representative density plots show the viability of HCT116 (**A**) and MCF-7 (**B**) cells following exposure to the indicated experimental conditions. Cells were incubated with magnetic nanoparticles at either 0.4 mg $_{Fe}$ mL^{-1} (C0.4) or 1.0 mg $_{Fe}$ mL^{-1} (C1.0) and either exposed or not to a static magnetic field (MF) gradient (≈350 mT) generated by Nd-Fe-B permanent magnets positioned beneath the culture plates (Control$_{+MF}$ and C1.0$_{+MF}$). Where indicated, samples were subsequently exposed to an alternating magnetic field (AMF; H = 23.9 kA m^{-1}, f = 228 kHz, t = 30 min). The lower left quadrant of each plot corresponds to viable (Annexin V^{-}/PI^{-}) cells, and the percentage of viable cells is indicated in each panel. Plots are representative of two independent biological replicates.*

Table S3. Summary of cell death analysis by Annexin V/PI staining. *Percentage of viable (Annexin V^-/PI^-), early apoptotic (Annexin V^+/PI^-), late apoptotic (Annexin V^+/PI^+), and necrotic (Annexin V^-/PI^+) cells in **(A)** HCT116 and **(B)** MCF-7 cell lines following 24 h incubation under the indicated experimental conditions (Fig. S4), as assessed by Annexin V/PI staining and flow cytometry. Data are presented as mean ± standard deviation from two independent experiments.*

(A) HCT116 cell line Group	$Annexin^-/PI^-$ (Q1: %)	$Annexin^+/PI^-$ (Q2: %)	$Annexin^+/PI^+$ (Q3: %)	$Annexin^-/PI^+$ (Q4: %)
Control	100.0 ± 0.0	0.0 ± 0.0	0.0 ± 0.0	0.0 ± 0.0
Control + AMF	96.0 ± 0.9	1.0 ± 0.3	2.0 ± 0.9	1.0 ± 0.2
C0.4	92.0 ± 1.5	2.0 ± 0.9	6.0 ± 0.3	0.0 ± 0.0
C0.4 + AMF	89.0 ± 0.9	1.0 ± 0.4	9.0 ± 2.3	1.0 ± 0.5
$C0.4_{+MF}$	91.0 ± 1.2	3.0 ± 0.8	4.0 ± 0.7	2.0 ± 1.1
$C0.4_{+MF}$ + AMF	56.0 ± 0.0	20.0 ± 0.3	23.0 ± 0.7	1.0 ± 0.4
C1.0	83.0 ± 1.1	7.0 ± 1.5	7.0 ± 0.0	3.0 ± 0.4
C1.0 + AMF	80.0 ± 0.5	11 ± 0.9	3.0 ± 0.1	6.0 ± 0.3
**(B) MC7 cell line Group**	**$Annexin^-/PI^-$ (Q1: %)**	**$Annexin^+/PI^-$ (Q2: %)**	**$Annexin^+/PI^+$ (Q3: %)**	**$Annexin^-/PI^+$ (Q4: %)**
Control	98.0 ± 0.9	2.0 ± 1.2	0.0 ± 0.0	0.0 ± 0.0
Control + AMF	95.0 ± 2.6	5.0 ± 3.7	0.0 ± 0.0	0.0 ± 0.0
C0.4	96.0 ± 1.9	4.0 ± 2.2	0.0 ± 0.0	0.0 ± 0.0
C0.4 + AMF	91.0 ± 3.5	9.0 ± 1.2	0.0 ± 0.0	0.0 ± 0.0
$C0.4_{+MF}$	91.0 ± 2.4	9.0 ± 3.1	0.0 ± 0.0	0.0 ± 0.0
$C0.4_{+MF}$ + AMF	41.0 ± 1.3	29.0 ± 3.4	16.0 ± 0.1	14.0 ± 4.8
C1.0	95.0 ± 0.7	5.0 ± 0.4	0.0 ± 0.0	0.0 ± 0.0
C1.0 + AMF	92.0 ± 0.6	3.0 ± 0.5	4.0 ± 0.4	1.0 ± 0.3

Table S4. Differences in cell viability between experimental groups treated with varying amounts of MNPs following exposure to an AMF in (A) HCT116 and (B) MCF-7 cell lines. *Statistical differences were assessed by three-way analysis of variance (ANOVA). Significance levels are indicated as follows: ****p < 0.0001; ***p < 0.001; **p < 0.01; *p < 0.05; p > 0.05 denotes no significant difference (n.s.). The row factor corresponds to percentage viability.*
The effects analysed were:
– Magnetic hyperthermia treatment effect: (AB vs CD), (a1B vs c1D), (Aa1 vs Cc1);
– Amount of magnetic nanoparticle (MNP) uptake effect: (AC vs BD), (a1c1 vs BD), (AC vs a1c1).
Experimental groups are defined as follows:
A/a1 = C0.4 / $C0.4_{+MF}$ (static magnetic field gradient only);
B = C1.0;
C/c1 = C0.4 + AMF / $C0.4_{+MF}$ + AMF;
D = C1.0 + AMF.

(A) HCT116 cell line (Row factor) = % viability	Groups			
	**A/ a1**	**B**	**C / c1**	**D**
Source of Variation	*% of total variation*	*P value*	*P value summary*	*Significant?*
(Row factor) x (AB vs CD) x (AC vs BD)	*0.0103*	*0,2633*	*ns*	*No*
(Row factor) x (a1B vs c1D) x (a1c1 vs BD)	*6.145*	*<0.0001*	******	*Yes*
(Row factor) x (Aa1 vs Cc1) x (AC vs a1c1)	*5.128*	*<0.0001*	******	*Yes*
**(B) MC7 cell line (Row factor) = % viability**	**Groups**			
	**A/ a1**	**B**	**C / c1**	**D**
Source of Variation	*% of total variation*	*P value*	*P value summary*	*Significant?*
(Row factor) x (AB vs CD) x (AC vs BD)	*0.04423*	*0.0469*	***	*Yes*
(Row factor) x (a1B vs c1D) x (a1c1 vs BD)	*9.992*	*<0.0001*	******	*Yes*
(Row factor) x (Aa1 vs Cc1) x (AC vs a1c1)	*8.313*	*<0.0001*	******	*Yes*
